# Multiple endocrine neoplasia type 2A syndrome presenting with corneal nerve thickening

**DOI:** 10.1093/qjmed/hcad259

**Published:** 2023-11-15

**Authors:** I Petrie, N Knox Cartwright, H Roberts, E Kyrodimou, C Moudiotis, M Owens, R Cleaver, J Smith, B Vaidya

**Affiliations:** Department of ENT, Royal Devon University Hospital, Exeter, UK; Department of Ophthalmology, Royal Devon University Hospital, Exeter, UK; Department of Ophthalmology, Royal Devon University Hospital, Exeter, UK; University of Exeter Medical School, Exeter, UK; Department of Pathology, Royal Devon University Hospital, Exeter, UK; Department of Paediatrics, Royal Devon University Hospital, Exeter, UK; Exeter Genomics Laboratory, Royal Devon University Hospital, Exeter, UK; Peninsula Clinical Genetics Service, Royal Devon University Hospital, Exeter, UK; Department of ENT, Royal Devon University Hospital, Exeter, UK; University of Exeter Medical School, Exeter, UK; Department of Endocrinology, Royal Devon University Hospital, Exeter, UK

Learning points for clinicians

•Corneal nerve thickening (CNT) is one of the characteristic phenotypic features associated with multiple endocrine neoplasia (MEN) type 2B, a syndrome characterized by aggressive medullary thyroid carcinoma and phaeochromocytoma.
•This case demonstrates that CNT is not exclusively associated with MEN2B and can also be a presenting feature of MEN2A.

## Introduction

Multiple endocrine neoplasia (MEN) type 2B is an autosomal dominant condition, typically associated with an early onset aggressive form of medullary thyroid carcinoma (MTC), phaeochromocytoma and distinctive phenotypic features including marfanoid habitus, mucosal neuroma and corneal nerve thickening (CNT). In contrast, MEN2A is associated with MTC, phaeochromocytoma and primary hyperparathyroidism but the additional phenotypic features seen in MEN2B are characteristically absent.^1^ We report a case presented to a local optician with CNT whose investigations led to the identification of a new lineage of MEN2A.

## Case presentation

**Figure 1.**
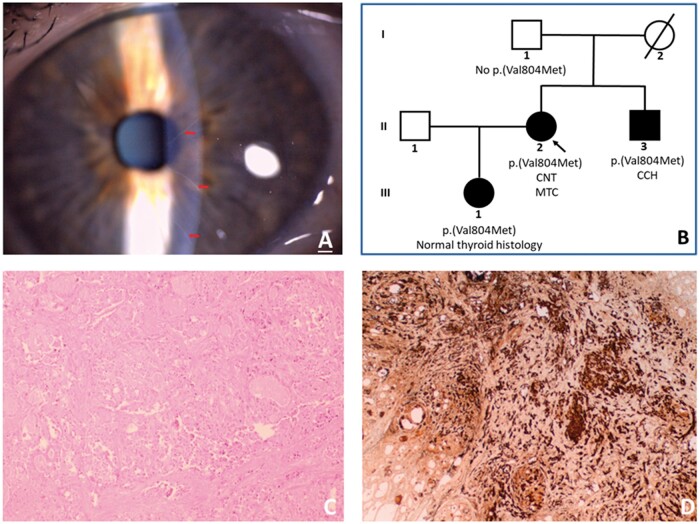
(**A**) Slit-lamp examination showing prominent corneal nerves (red arrows). (**B**) Pedigree of the family. Females are designated by circles, and males by squares. II.2 is the proband. Family members carrying pathogenic *RET* gene variant, p.(Val804Met) are indicated with blackened symbols. Histology following thyroidectomy showed medullary thyroid carcinoma (MTC) in II.2, C-cell hyperplasia (CCH) in II.3 and normal features in III.1. Corneal nerve thickening (CNT) was seen in II.2 but absent in II.3 and III.1. (**C**) Thyroid histology from the proband consistent with MTC (Haematoxylin & Eosin, magnification 10×). (**D**) Immunohistochemical staining for calcitonin in the thyroid specimen from the proband confirming MTC (magnification 4×).

A 46-year-old woman presented to her local optician with gritty eyes and was found to have CNT (Figure 1A). This prompted a referral to endocrinology through ophthalmology to investigate a possible diagnosis of MEN2B. Her past medical history included Hashimoto’s thyroiditis. Her mother, who died at age 60, had a history of an unspecified ‘thyroid problem’ (Figure 1B). Apart from CNT, the proband had a high-arched palate but no other phenotypic features of MEN2B. She had no palpable goitre or lymphadenopathy. Her genetic testing did not identify pathogenic variants p.(Ala883Phe) and p.(Met918Thr) associated with MEN2B in exons 15 and 16 of the *RET* gene (NM_ 020975.4),^1^ or any pathogenic variants in exon 20 of the *SOS1* gene (NM_ 005633.4), previously we found to be associated with mucosal neuroma syndrome and CNT.^2^ However, her biochemistry test revealed an elevated calcitonin (201 ng/l; reference range ≤4.8), raising a suspicion of MTC. Serum calcium, serum parathyroid hormone and plasma normetanephrine and metanephrine levels were normal. Thyroid ultrasound showed a heterogenous hypoechoic appearance with multiple small nodules, consistent with the diagnosis of Hashimoto’s thyroiditis. The largest nodule was 7 mm and was associated with microcalcification, and its fine needle aspiration showed benign follicular cells (THY2). Subsequent extended *RET* gene testing (exons 1–20) identified a heterozygous pathogenic variant in exon 14, c.2410G>A p.(Val804Met), associated with moderate risk MEN2A.^1^ She underwent total thyroidectomy and central neck dissection. Histology indicated bilateral MTC with lympho-vascular invasion and multiple central node metastases (pT1a pN1a M0) (Figure 1C and D) as well as an incidental sub-centimetre papillary carcinoma in a background of extensive Hashimoto’s thyroiditis.

Cascade genetic testing in her family members identified the heterozygous *RET* gene pathogenic variant in the proband’s daughter (aged 12 years) and brother (aged 39 years) (Figure 1B). Both relatives underwent a total thyroidectomy. The proband’s daughter’s histology was normal, whereas the brother’s histology showed c-cell hyperplasia and two foci of incidental sub-centimetre papillary carcinoma. Slit-lamp examination showed neither relative had CNT.

## Discussion

CNT is one of the distinctive phenotypic features associated with MEN2B, and its finding warrants investigation for this potentially life-threatening syndrome.^1^^,^^3^ We rapidly excluded MEN2B in our proband with genetic testing, although a diagnosis of MEN2B would have been unusual given her age at presentation, as, in this condition, an aggressive form of MTC often manifests in infancy or early childhood. Her subsequent investigations led to the diagnosis of MEN2A.

Although an association between CNT and MEN2A is less well documented, a few previous studies have reported the presence of CNT in patients with MEN2A.^4–6^ When a cohort of patients with MEN2A was examined with slit-lamp systematically, over a quarter (6 out of 22) of the patients were found to have grade 3–4 prominent corneal nerves.^4^ As CNT is often asymptomatic and requires slit-lamp examination to detect, it is thought to be underreported in MEN2A.

The *RET* gene encodes a transmembrane receptor, tyrosine kinase which has an important role in the development of neuro-endocrine tissues. Thus, the gain of function *RET* gene pathogenic variants associated with MEN2A and MEN2B can lead to the development of endocrine tumours, such as MTC, and abnormal neuronal growth, including CNT. It is interesting to note that our proband’s brother and daughter did not have CNT despite sharing the same pathogenic *RET* variant. Similar intrafamilial variation in the presence and the degree of CNT in families with MEN2A has been reported previously.^4^^,^^6^ In conclusion, our case together with the previous observations highlights that, although a characteristic feature of MEN2B, CNT is not exclusive to this syndrome, and its finding should raise suspicion of both MEN2B and MEN2A.

## Funding

This study was not supported by any funding.

## Conflict of interest

None declared.
